# Alum Activates the Bovine NLRP3 Inflammasome

**DOI:** 10.3389/fimmu.2017.01494

**Published:** 2017-11-09

**Authors:** Ciaran Harte, Aoife L. Gorman, S. McCluskey, Michael Carty, Andrew G. Bowie, C. J. Scott, Kieran G. Meade, Ed C. Lavelle

**Affiliations:** ^1^Adjuvant Research Group, School of Biochemistry and Immunology, Trinity College Dublin, Trinity Biomedical Sciences Institute, Dublin, Ireland; ^2^Animal and Bioscience Research Department, Animal and Grassland Research and Innovation Centre, Teagasc, Grange, Ireland; ^3^Viral Immune Evasion Group, School of Biochemistry and Immunology, Trinity College Dublin, Trinity Biomedical Sciences Institute, Dublin, Ireland; ^4^Molecular Therapeutics, School of Pharmacy, Queen’s University Belfast, Belfast, United Kingdom

**Keywords:** adjuvant, alum, bovine, IL-1, inflammasome, peripheral blood mononuclear cells, vaccine

## Abstract

There has been a move away from vaccines composed of whole or inactivated antigens toward subunit-based vaccines, which although safe, provide less immunological protection. As a result, the use of adjuvants to enhance and direct adaptive immune responses has become the focus of much targeted bovine vaccine research. However, the mechanisms by which adjuvants work to enhance immunological protection in many cases remains unclear, although this knowledge is critical to the rational design of effective next generation vaccines. This study aimed to investigate the mechanisms by which alum, a commonly used adjuvant in bovine vaccines, enhances IL-1β secretion in bovine peripheral blood mononuclear cells (PBMCs). Unlike the case with human PBMCs, alum promoted IL-1β secretion in a subset of bovine PBMCs without priming with a toll-like receptor agonist. This suggests that PBMCs from some cattle are primed to produce this potent inflammatory cytokine and western blotting confirmed the presence of preexisting pro-IL-1β in PBMCs from a subset of 8-month-old cattle. To address the mechanism underlying alum-induced IL-1β secretion, specific inhibitors identified that alum mediates lysosomal disruption which subsequently activates the assembly of an NLRP3, ASC, caspase-1, and potentially caspase-8 containing complex. These components form an inflammasome, which mediates alum-induced IL-1β secretion in bovine PBMCs. Given the demonstrated role of the NLRP3 inflammasome in regulating adaptive immunity in murine systems, these results will inform further targeted research into the potential of inflammasome activation for rational vaccine design in cattle.

## Introduction

IL-1β, a member of the IL-1 cytokine family, is an inflammatory cytokine that mediates an array of effector functions including vasodilation, inflammatory cell infiltration, and adhesion molecule expression (1). Primarily produced by monocytes, macrophages, and dendritic cells, IL-1β synthesis and secretion is tightly regulated. It requires a signal (“signal 1”), generally in the form of a pathogen-associated molecular pattern (PAMP) or endogenous danger signal to induce the expression of pro-IL-1β, followed by “signal 2” to activate caspase-1, which processes the cytokine into its active form (2, 3). The NLRP3 inflammasome, comprising NLRP3, ASC, and caspase-1, is the best characterized inflammasome and can be activated by a range of stimuli including uric acid, cholesterol crystals, ATP, silica, and asbestos (4). Additionally, in murine and human cells it has been shown that alum based adjuvants can activate the NLRP3 inflammasome and caspase-1, resulting in the secretion of bioactive IL-1β (5).

Presently, alum is one of a select few adjuvants approved for use in human and veterinary vaccines. Despite its widespread use, the immuno-modulatory properties of alum are not fully understood and have received little attention in a bovine context. Upon injection, alum recruits an array of innate cells including monocytes, dendritic cells, NK cells, and neutrophils (5–7). Additionally, alum injection triggers the production of numerous inflammatory cytokines and chemokines including IL-1β, IL-18, and keratinocyte chemoattractant (6, 8). In mice, it has been established that alum-induced IL-1β secretion is reduced in NLRP3 and caspase-1-deficient cells *in vitro*. Alum is an effective adjuvant at promoting antigen specific humoral immunity but has limited capacity to promote Th1 responses (6, 9–11). As result, alum is not an optimal adjuvant for all vaccines. However, many of these findings are based on murine studies so understanding the mechanism by which alum enhances immune responses in a bovine context is important to advance bovine vaccine development.

Interindividual variation in responses to both antigens and adjuvants may contribute to suboptimal efficacy of a number of bovine vaccines. These systems may be developed in mouse models and are limited by the lack of a sufficiently detailed understanding of the bovine immune response and therefore often do not work well in cattle (12). Given the significant differences in innate immunity between species and the widespread use of alum as an adjuvant in cattle, it is therefore important to address the specific effects of alum in bovine cells.

## Materials and Methods

### Ethics Statement

All animal procedures were carried out according to the provisions of the EU Protection of Animals Used for Scientific Purposes Regulations 2012 (SI No. 543 of 2012) as amended and Directive 2010/63/EU of the European Parliament issued from the Health Products Regulatory Authority Ireland—license number AE 19132/P030. Human blood samples were collected from anonymous healthy blood donors from the Irish Blood Transfusion Service (IBTS) under license number (BI-AG-300919) issued from the School of Biochemistry and Immunology Research Ethics Committee, Trinity College Dublin in accordance with the Declaration of Helsinki.

### Animals

All animals used in this research project were healthy Holstein-Friesian calves under 1 year old (unless stated). Buffy coats were collected from anonymous healthy human blood donors.

### Reagents

The stimuli used to activate cells were LPS, *Escherichia coli* Serotype R515 (Enzo Life Sciences) and alhydrogel (Brenntag Biosector). Inhibitory molecules used in this research included: MCC950 (Cayman Chemical), caspase1-Z-YVAD-FMK (Bachem), caspase8-Z-IETD-FMK (Bachem), CA-074 (Sigma-Aldrich), and cathepsinB-CA-074-Me (Sigma-Aldrich). Antibodies used for western blotting included: polyclonal anti bovine IL-1β (Bio-Rad), polyclonal (N-15-R) antimouse ASC (Santa Cruz Biotechnology sc-22514-R), and monoclonal (AC-74) β-actin (Sigma-Aldrich). ELISA kits used to detect bovine IL-1β and human IL-1β were sourced from ThermoScientific and R&D Systems, respectively. The FLICA™ Assay Kit (FAM-YVAD-FMK) for caspase-1 detection was acquired from ImmunoChemistry Technologies.

### Peripheral Blood Mononuclear Cell (PBMC) Isolation and Culture

Bovine PBMCs were isolated from whole blood samples collected in 9 ml vacutainers containing Heparin anticoagulant. Human PBMCS were extracted from buffy coats. PBMCs were isolated using leucosep tubes (Greiner Bio-One, Storehouse, UK) and a density gradient histopaque 1077 (Sigma-Aldrich). Red blood cell contamination was eliminated using sterile 0.25% sodium chloride (Baxter) as a lysis buffer. The cells were subsequently centrifuged twice in PBS at a speed of 400 g for 10 min. For innate cytokine analysis, cells were incubated at 37°C with 5% CO_2_ in RMPI 1640 medium (Biosera) enriched with heat-inactivated fetal calf serum (Biosera), l-glutamine (Gibco), and penicillin (Gibco).

### ELISA

Supernatants from treated cells were used to measure IL-1β secretion by ELISA as per the manufacturer’s protocols. Absorbance was read on a Multiscan FC plate reader and analyzed with SkanIt for Multiscan FC software (Thermo Scientific). The limit of detection was between 31.25 and 2,000 pg/ml.

### Western Blotting

Peripheral blood mononuclear cells were lysed using 100 µl of Laemmli buffer (4% SDS, 10% 2-mercaptoethanol, 20% glycerol, 0.004% bromophenol blue, 0.125 M Tris–HCl). The lysates were transferred onto a 0.2 µm PVDF membrane (Millipore) and probed with anti-IL-1β, anti-ASC, and anti-β-actin antibodies. The blots were developed using a Bio-Rad ChemiDoc Imaging system (Bio-Rad).

### FLICA™ Assay

In preparation for caspase-1 analysis, cells were seeded at a density of 1 × 10^7^/ml and incubated with stimuli. Following incubation, the cells were resuspended and then centrifuged at 1,200 rpm for 5 min to pellet the cells. The supernatants were discarded and the cells were resuspended in FACs buffer (1% FCS in PBS) and incubated for 30 min with caspase-1 specific probe. The cells were centrifuged and washed three times in FACs buffer and analyzed by flow cytometry.

### Confocal Microscopy

Peripheral blood mononuclear cells (0.5 × 10^6^ cells/ml) were plated in cRPMI on 35 mm glass bottom tissue dishes. Cells were treated with alum (50 µg/ml), LPS (1 pg/ml), alum + LPS, and or RPMI only and stained with calcein as outlined by Khormaee et al. (13). Cells were viewed using a Point Scanning Confocal Microscope with a heated stage and CO_2_ chamber (Olympus FV100 LSM Confocal Microscope).

### Statistics

Statistical analysis was performed using Graphpad Prism 5 software. The means for two groups were compared using a paired *T*-test. The means for three or more groups were compared using one-way ANOVA. A *p*-value of <0.05 was taken as statistically significant.

## Results

### Alum Promotes IL-1β Secretion by Bovine PBMCs

**Figure 1:**
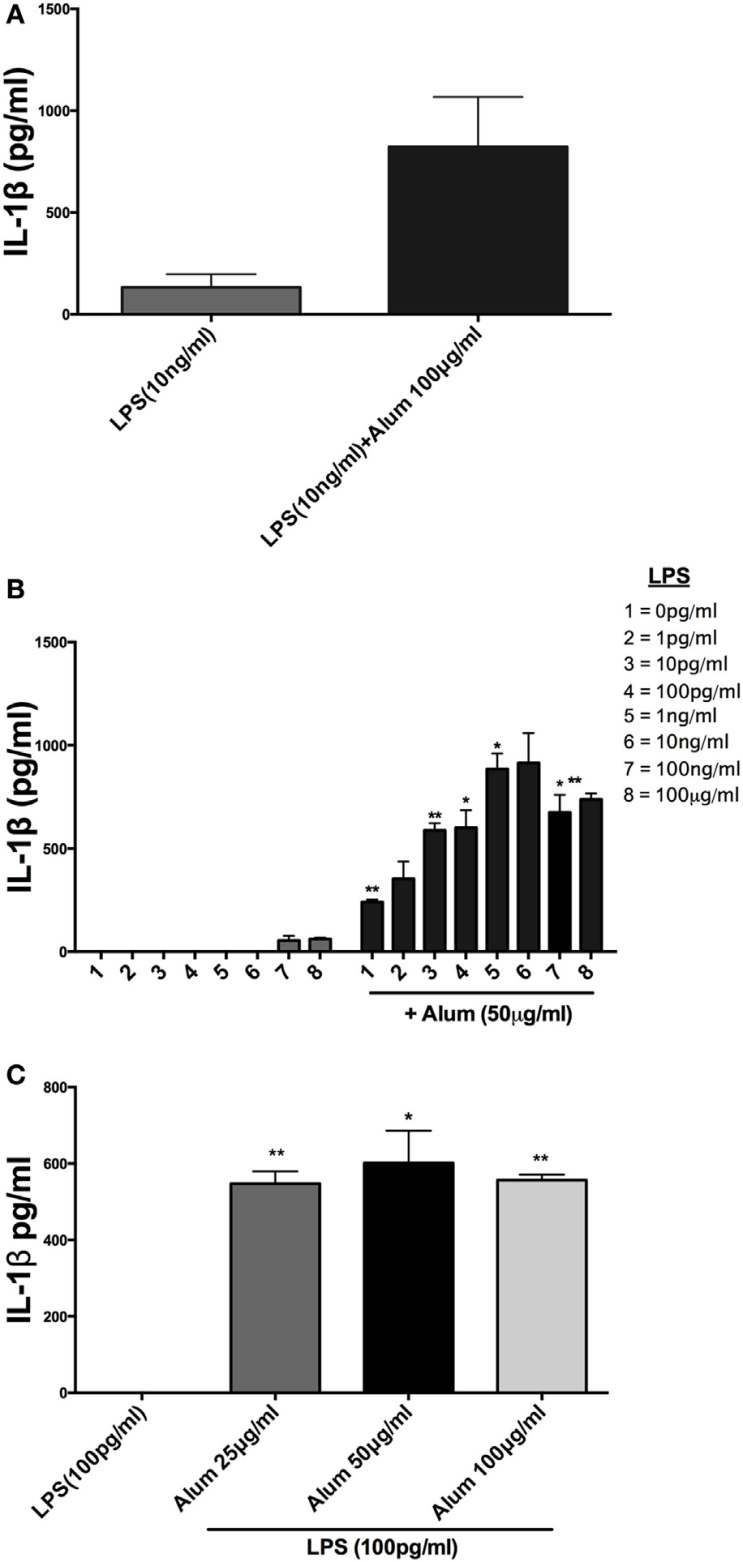
Alum enhances IL-1β secretion by bovine peripheral blood mononuclear cells (PBMCs). Cells (0.625 × 10^6^ cells/ml) from three animals were stimulated with RPMI, LPS, and/or alum for 24 h and supernatants were tested for IL-1β using ELISA. PBMCs were primed with LPS (10 ng/ml) for 3 h before the cells were stimulated with alum (100 µg/ml) for 24 h **(A)**. Cells were primed with concentrations of LPS ranging from 1 pg/ml to 1 μg/ml and a fixed dose of alum (50 µg/ml) **(B)**. PBMCs were primed with a single dose of LPS (100 pg/ml) and alum at a concentration of 25, 50, or 100 µg/ml **(C)**. For ELISA analysis, results are mean cytokine concentrations (+SEM) in supernatants that were tested individually in triplicate. *p < 0.05 and **p < 0.01 were calculated using GraphPad.

It has been previously established in murine and human cells that a combination of LPS and alum effectively drives NLRP3 inflammasome dependent IL-1β secretion (14), although this has not been investigated in cattle. Bovine PBMCs were isolated from Friesian calves (<6 months old) and treated with a range of LPS and alum concentrations for 24 h. Bovine PBMCs secreted small amounts of IL-1β in response to LPS (10 ng/ml) but secretion was significantly elevated when alum (100 µg/ml) was added (Figure 1A).

**Figure 2:**
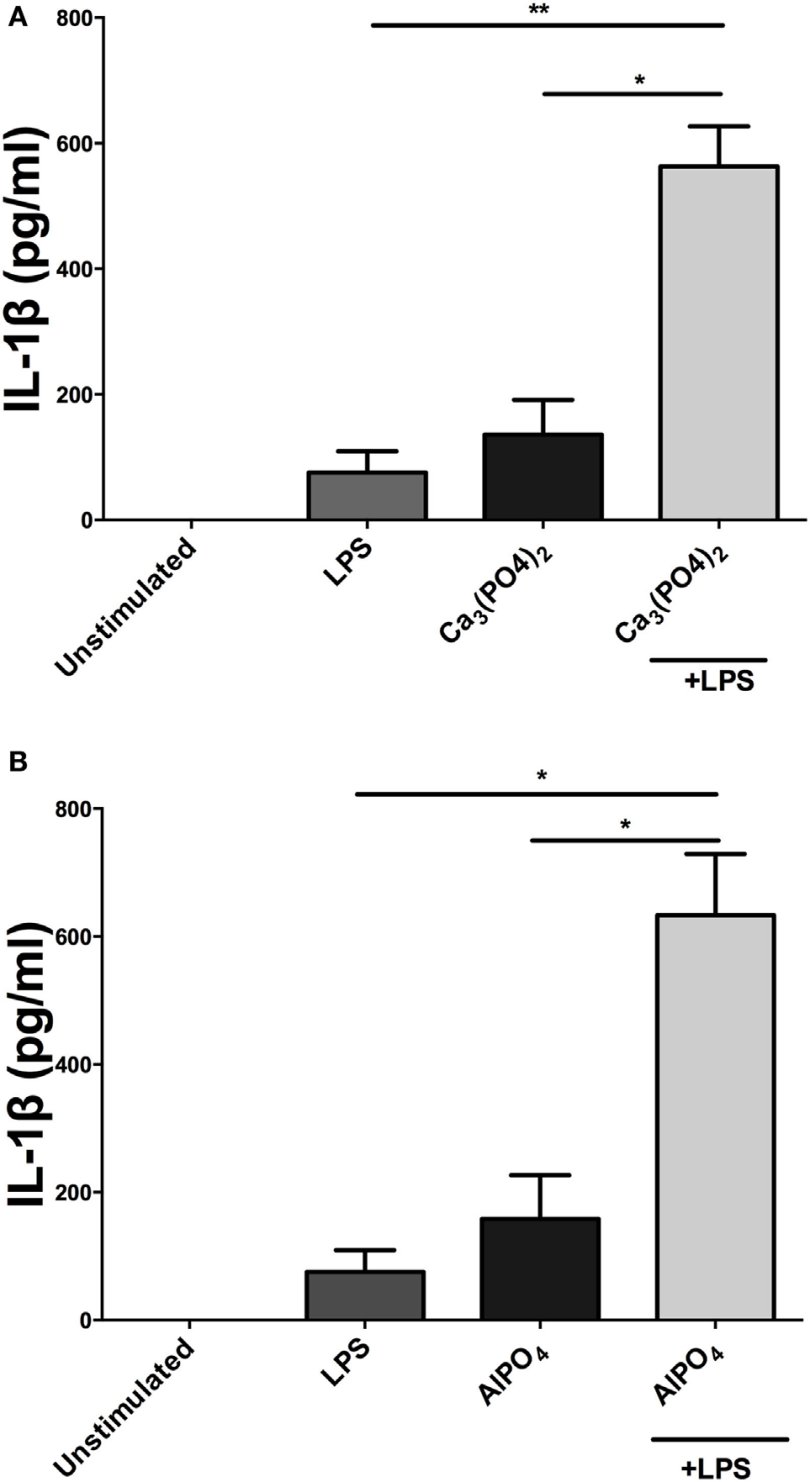
Calcium phosphate and aluminum phosphate adjuvants enhance IL-1β secretion by bovine peripheral blood mononuclear cells (PBMCs). Cells (0.625 × 10^6^ cells/ml) from three animals were stimulated with RPMI, LPS, and/or calcium phosphate **(A)** or aluminum phosphate **(B)** for 24 h and supernatants were tested for IL-1β using ELISA. PBMCs were primed with LPS (10 ng/ml) for 3 h before cells were stimulated with adjuvants over a range of concentrations for 24 h. For ELISA analysis, results are mean cytokine concentrations (+SEM) in supernatants that were tested individually in triplicate. *p < 0.05 and **p < 0.01 were calculated using GraphPad.

Incubation of PBMCs with a fixed dose of alum (50 µg/ml) and LPS concentrations from 1 pg/ml to 1 µg/ml resulted in higher IL-1β secretion than the corresponding amounts of LPS alone (Figure 1B). A surprising observation was that cells stimulated with alum in the absence of LPS secreted IL-1β (Figure 1B). This observation contrasts what is seen in murine and human macrophages and dendritic cells. A more detailed analysis found that addition of alum to cells at 25, 50, or 100 µg/ml enhanced IL-1β secretion from LPS-primed PBMCs (Figure 1C). To address whether the IL-1β promoting ability of alhydrogel also applied to aluminum phosphate and calcium phosphate, PBMCs were treated with these clinically applied adjuvants alone or following priming with LPS. As seen with alhydrogel, both calcium phosphate and aluminum phosphate were potent stimuli for promoting IL-1β secretion by bovine cells (Figures 2A,B).

### PBMCs from a Subset of Calves Contain Preformed PRO-IL-1β

**Figure 3:**
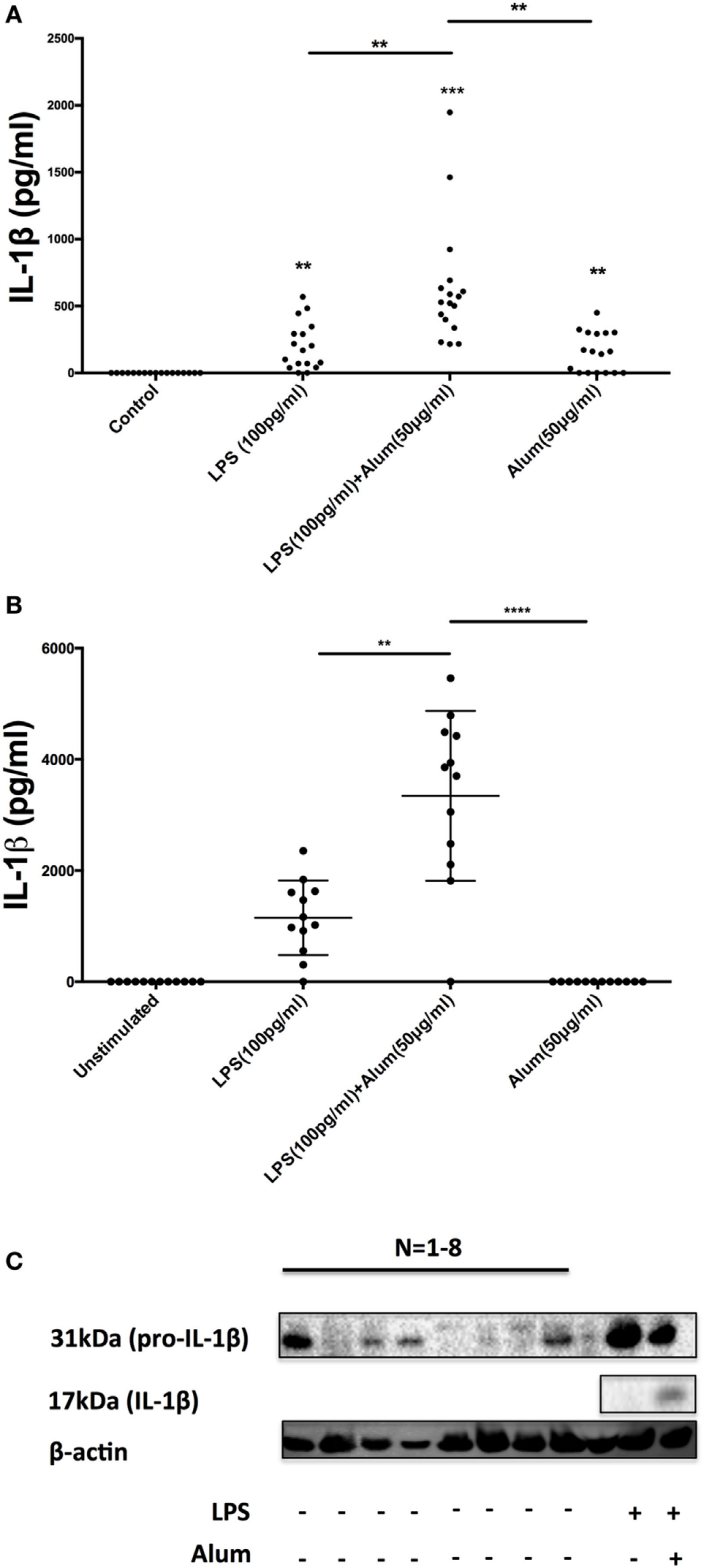
A subset of bovine, but not human, peripheral blood mononuclear cells (PBMCs) contain preformed IL-1β. Bovine PBMCs from 17 Friesian cows were stimulated with LPS (100 pg/ml) and alum (50 µg/ml) for 24 h and tested for IL-1β secretion by ELISA **(A)**. PBMCs from 12 human donors were treated with LPS (100 pg/ml) and alum (50 µg/ml) and tested for IL-1β using ELISA **(B)**. Bovine cells (2 × 10^6^ cells/ml) from eight cows were left unstimulated or treated with LPS and or/alum for 6 h and analyzed by western blot for pro-IL-1β **(C)**. For ELISA analysis, results are mean cytokine concentrations (+SEM) in supernatants that were tested individually in triplicate. *p < 0.05, **p < 0.01, ***p < 0.001 were calculated using GraphPad.

To secrete IL-1β, innate immune cells generally require two signals in the form of a PAMP agonist and an inflammasome activator (15, 16). Typically, toll-like receptor (TLR) agonists prime cells to synthesize inactive pro-IL-1β while activation of the inflammasome triggers caspase-1 activation and the cleavage and secretion of processed IL-1β. Given the remarkable observation that alum could trigger the secretion of IL-1β in the absence of priming with LPS (Figure 1B), it was hypothesized that PBMCs from some calves may contain preformed pro-IL-1β and as such require no TLR priming. To address this, PBMCs were isolated from 17 calves (aged < 1 year old) and were treated with LPS, alum or a combination of LPS and alum for 24 h. The control cells were left unstimulated. Cells stimulated with LPS and alum together for 24 h secreted high concentrations of IL-1β (Figure 3A). Moreover, PBMCs from 10 of 17 animals secreted IL-1β when incubated with alum in the absence of LPS (Figure 3A).

To address whether this response was bovine specific, PBMCs from 12 humans were isolated and treated with LPS (100 pg/ml) and alum (50 µg/ml). In contrast to the findings with bovine PBMCs there was no detectable secretion of IL-1β by human PBMCs following incubation with alum when LPS was absent (Figure 3B). However, in response to LPS and alum combined, human PBMCs secreted high concentrations of IL-1β (Figure 3B).

To determine whether alum drives the expression of pro-IL-1β as well as IL-1β secretion, PBMCs were isolated from 8 calves and cultured in the presence or absence of LPS and/or alum for 6 h. The 31 kDa pro-IL-1β band was detected in cells stimulated with LPS (Figure 3C). Interestingly, in four of the eight animals tested, pro-IL-1β was also present in unstimulated cells thus suggesting that some bovine monocular cells are already primed to secrete IL-1β (Figure 3C).

### Alum-Induced IL-1β Secretion in Bovine PBMCs Is Caspase-1 Dependent

In humans, it has been demonstrated that alum-mediated IL-1β secretion is caspase-1 dependent (8). Conversely, although alum has been incorporated into numerous veterinary vaccines, its signaling capacity in a bovine context remains unknown. As a result, the role of caspase-1 in alum-induced bovine IL-1β secretion was next investigated.

**Figure 4:**
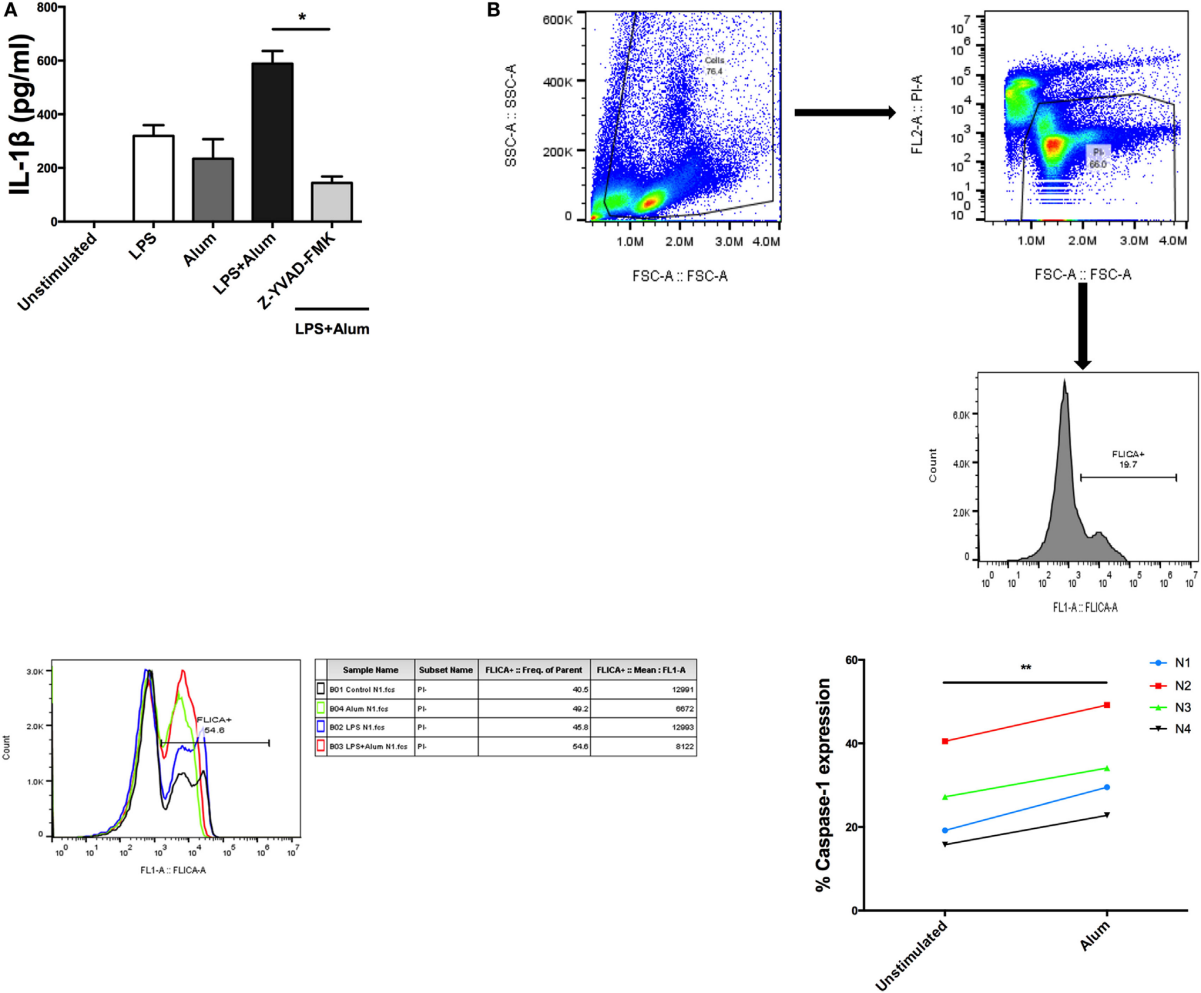
Alum-induced IL-1β secretion is caspase-1 dependent. LPS (100 pg/ml)-primed cells from three animals were incubated with Z-YVAD-FMK (10 µM) for 40 min. Following this, the cells were stimulated with alum (50 µg/ml) for 24 h and supernatants were then harvested and analyzed by ELISA for IL-1β **(A)**. Cells were seeded (1.5 × 10^6^) and treated with LPS and alum for 2 h. Following this, the cells were analyzed by flow cytometry to assay for caspase-1 **(B)**. The data are representative of four animals (<1 year old) **(B)**. For ELISA analysis, results are mean cytokine concentrations (+SEM) in supernatants that were tested individually in triplicate. *p < 0.05, **p < 0.01, ***p < 0.001 were calculated using GraphPad.

When bovine PBMCs were incubated with a caspase-1 inhibitor, Z-YVAD-FMK (10 µm), prior to addition of alum, and after stimulation with LPS, a substantial reduction in IL-1β secretion was observed (Figure 4A). To further prove that caspase-1 is involved in alum-induced IL-1β secretion, cells from four animals were stimulated with cRPMI or alum. Activated caspase-1 was detected using a caspase-1 detection probe termed FLICA 660-YVAD-FMK. This probe is comprised of an affinity peptide sequence specific for caspase-1 (YVAD), a far-red fluorescent 660 dye and a fluoromethyl ketone reactive moiety. This reagent enters cells and irreversibly binds to active caspase-1 thereby emitting and retaining the red signal inside cells positive for caspase-1. The results were subsequently analyzed by flow cytometry. The results demonstrate that the percentage of cells positive for caspase-1 activity is increased in response to alum (Figure 4B).

### Alum-Induced IL-1β Secretion in Bovine PBMCs Is NLRP3 and Caspase-8 Dependent

In mice, it has been demonstrated that alum-mediated IL-1β secretion is NLRP3 dependent (14, 17). The role of caspase-8 in alum-induced IL-1β responses has received less attention. Recent publications have indicated that in response to TLR4 activation, caspase-8 is required for NLRP3 inflammasome-dependent IL-1β processing in murine cells (18). The role of NLRP3 and caspase-8 in alum-induced bovine IL-1β secretion has not been previously investigated.

**Figure 5:**
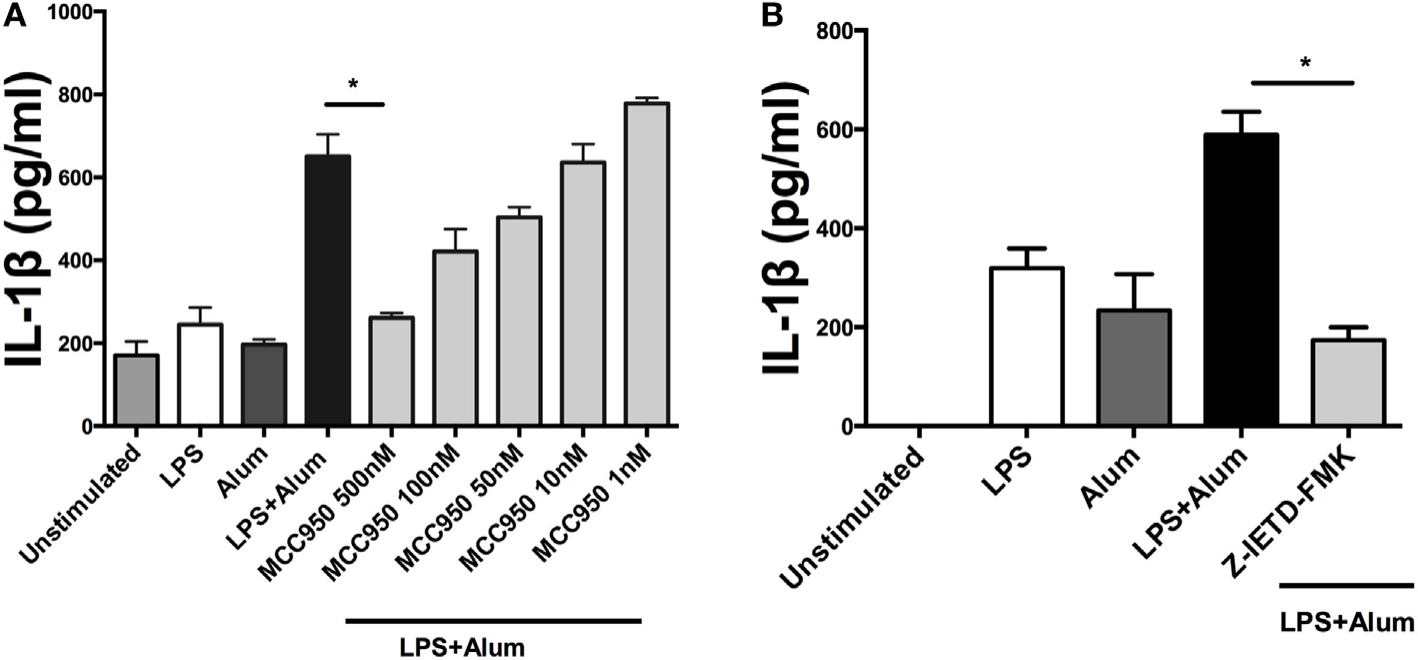
Alum-induced IL-1β secretion is NLRP3 and caspase-8 dependent. LPS (100 pg/ml)-primed cells from three animals were incubated with MCC950 (500, 100, 50, 10, and 1 nM) **(A)** and Z-IETD-FMK (10 µm) **(B)**. After 40 min, the cells were stimulated with alum (50 µg/ml) for 24 h and supernatants were then harvested and analyzed by ELISA for IL-1β. For ELISA analysis, results are mean cytokine concentrations (+SEM) in supernatants that were tested individually in triplicate. *p < 0.05, **p < 0.01, ***p < 0.001 were calculated using GraphPad.

Having established that alum-induced IL-1β secretion was caspase-1 dependent in bovine cells, the potential role of the NLRP3 inflammasome was addressed. LPS-primed PBMCs were pretreated with MCC950, a specific NLRP3 inflammasome inhibitor (19), prior to the addition of alum. After 24 h the supernatants were analyzed by ELISA for IL-1β secretion. LPS and alum stimulation induced significantly less IL-1β secretion from cells cocultured with 500 nM MCC950 (Figure 5A). In the presence of 100, 50, 10, and 1 nM of MCC950, alum-induced IL-1β secretion was not abrogated (Figure 5A).

To determine whether alum-induced IL-1β secretion is caspase-8 dependent, LPS-primed PBMCs were incubated with Z-IETD-FMK, a caspase-8 inhibitor, after which they were cocultured with alum for 24 h. IL-1β secretion was suppressed by caspase-8 inhibition after 24 h (Figure 5B). These data suggest that caspase-8 and caspase-1 both contributed to alum-induced IL-1β secretion by bovine PBMCs.

### Alum Promotes ASC Oligomerization

Inflammasome activation involves NLRP3 recruiting and binding to the adaptor protein ASC through interactions involving the PYD domain on both molecules (20). When bound to NLRP3, ASC oligomerizes to form a pyroptosome which then binds to pro-caspase-1 *via* homotypic interactions (21). The assembled inflammasome facilitates the cleavage and activation of caspase-1, which cleaves pro-IL-1β to its bioactive form resulting in its secretion from the cell (21, 22). In murine models, it has been demonstrated that ASC oligomerization is crucial for the activation of caspase-1 (22–24).

**Figure 6:**
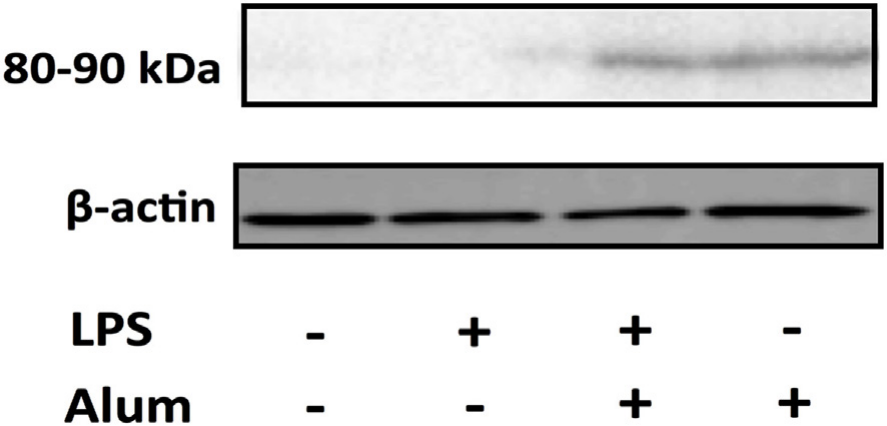
Alum promotes the oligomerization of ASC. Cells (10 × 10^6^) were pretreated with LPS 3 h before alum was administered. Cell lysates were harvested 3 h later and ASC oligomers (80–90 kDa) were detected through western blotting. The data are representative of two animals aged <6 months old.

To assess the effects of alum on ASC oligomerization in bovine cells, PBMCs were cultured at a density of 10^7^ /ml. Cells were left unstimulated, treated with LPS (100 pg/ml), alum (50 µg/ml), or a combination of both for 3 h. Afterward, the cells were lysed and probed for ASC oligomerization by western blotting. In response to alum alone or together with LPS, the oligomerization of ASC was evident by detection of an 80–90 kDa molecular weight band (Figure 6; Figure S3 in Supplementary Material). The oligomer was absent in unstimulated cells or those incubated with LPS alone.

### Cathepsin Activity Is Required for Alum-Induced IL-1β Secretion from Bovine PBMCS

**Figure 7:**
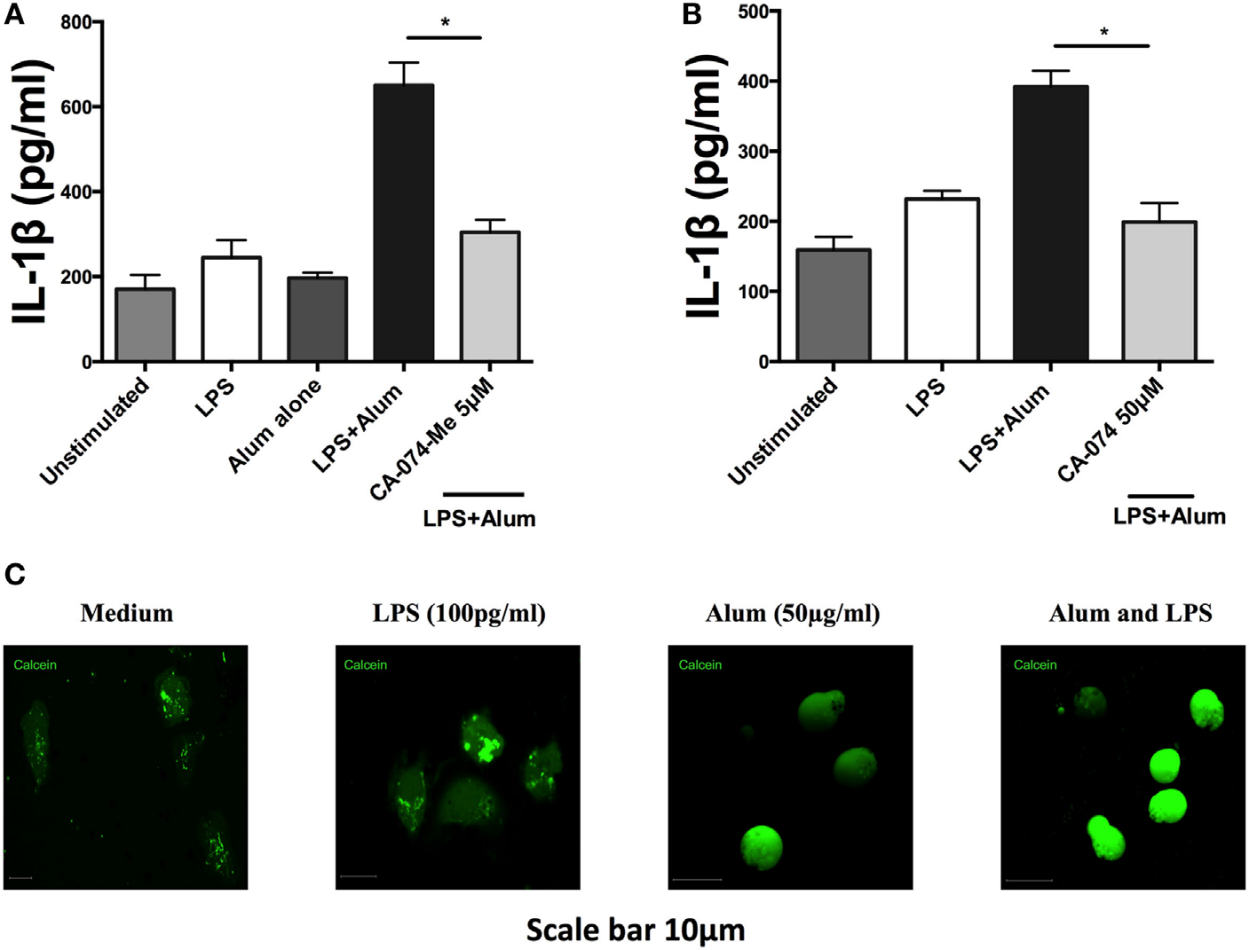
Alum induces lysosomal disruption and inhibition of lysosomal cathepsin B reduces alum-induced IL-1β secretion in bovine peripheral blood mononuclear cells (PBMCs). Bovine PBMCs from three animals were seeded and stimulated with LPS (100 pg/ml). Cells were then incubated with CA-074Me **(A)** or CA-074 **(B)** prior to stimulation with alum (50 µg/ml). Supernatants were collected after 24 h and IL-1β concentrations were detected by ELISA. **(C)** PBMCs treated with medium, LPS or alum were incubated with calcein for 3hr and visualized by confocal microscopy. For ELISA analysis, results are mean cytokine concentrations (+SEM) in supernatants that were tested individually in triplicate. *p < 0.05, **p < 0.01, ***p < 0.001 were calculated using GraphPad.

Studies on murine macrophages and dendritic cells have demonstrated that alum induces lysosomal disruption resulting in activation of the NLRP3 inflammasome (25, 26). Additionally, lysosomal damage and release of hydrolases has been shown to up-regulate caspase-1 activity (27). To determine whether lysosomal proteases play a role in regulating bovine IL-1β secretion, PBMCs were cultured for 1 h with an inhibitor (CA-074-Me) targeting cathepsin B, a protease released during lysosomal damage. Following that, the cells were stimulated with alum (50 µg/ml) for 24 h after which the supernatants were collected and tested for IL-1β. Cells stimulated with LPS and alum produced substantially less IL-1β when incubated in the presence of the cathepsin B inhibitor than those cultured without the inhibitor (Figure 7A). Since at high concentrations this inhibitor can also inhibit other lysosomal cysteine cathepsins (28), we confirmed these results with CA-074 which although less cell permeable than CA-074-Me, has been shown to have enhanced selectivity for cathepsin B (28) (Figure 7B). CA074 inhibited alum-induced IL-1β secretion at a concentration of 50 µM. Finally to confirm that alum results in lysosomal damage, we stained cells with calcein. Calcein is endocytosed by cells in a non-specific manner, but cannot diffuse across endosomal membranes. This leads to calcein becoming trapped in endosomes resulting in a “punctate” staining pattern (13), as observed when bovine PBMCs are cotreated with RPMI or LPS in Figure 7C. However, when taken up by PBMCs that have been costimulated with alum, calcein homogenously stains the entire cell as a result of alum-driven lysosomal disruption and subsequent leakage of lysosomal contents (including calcein) into the cell cytoplasm.

## Discussion

Inflammasomes are key intracellular signaling components that regulate immunity through activating caspase-1, which subsequently leads to the processing of the cytokines IL-1β and IL-18. It has been demonstrated that IL-1β is a key regulator of T-cell cytokine production, can restrict bacterial replication in macrophages (29), and recruits neutrophils to the site of infection (30–33). Although inflammasome activation has been implicated as an important component of the bovine immune response to pathogens, including mycobacteria (34), the detailed mechanisms of inflammasome activation in a bovine context have not been addressed.

Given the distinct evolutionary differences between species, mechanistic understanding from human and murine studies cannot be assumed to apply in other species, including cattle. For example, in contrast to humans and mice, genes that encode DNA sensing inflammasome-associated proteins like the Pyrin and Hin domain family are Pseudogenised in some mammals including cattle (35). This suggests that these mammals have evolved alternate mechanisms to cope with DNA viruses. Species-specific investigations are therefore warranted, particularly in order to enhance the efficacy of novel vaccination strategies (36).

In accordance with observations made in murine and human studies, results from this study demonstrate that alum enhances IL-1β secretion from bovine PBMCs. Surprisingly, in the absence of LPS, alum was sufficient for promoting IL-1β secretion from PBMCs in a subset of animals, a result in contrast to what is observed in murine and human macrophages (14, 37). These results show for the first time that IL-1β activation in PBMCs from a subset of animals is not solely dependent on LPS stimulation and that a subset of Friesian cattle have primed cells capable of secreting IL-1β in response to alum alone. Moreover, this was not seen in human PBMCs suggesting a bovine-specific response.

**Figure 8:**
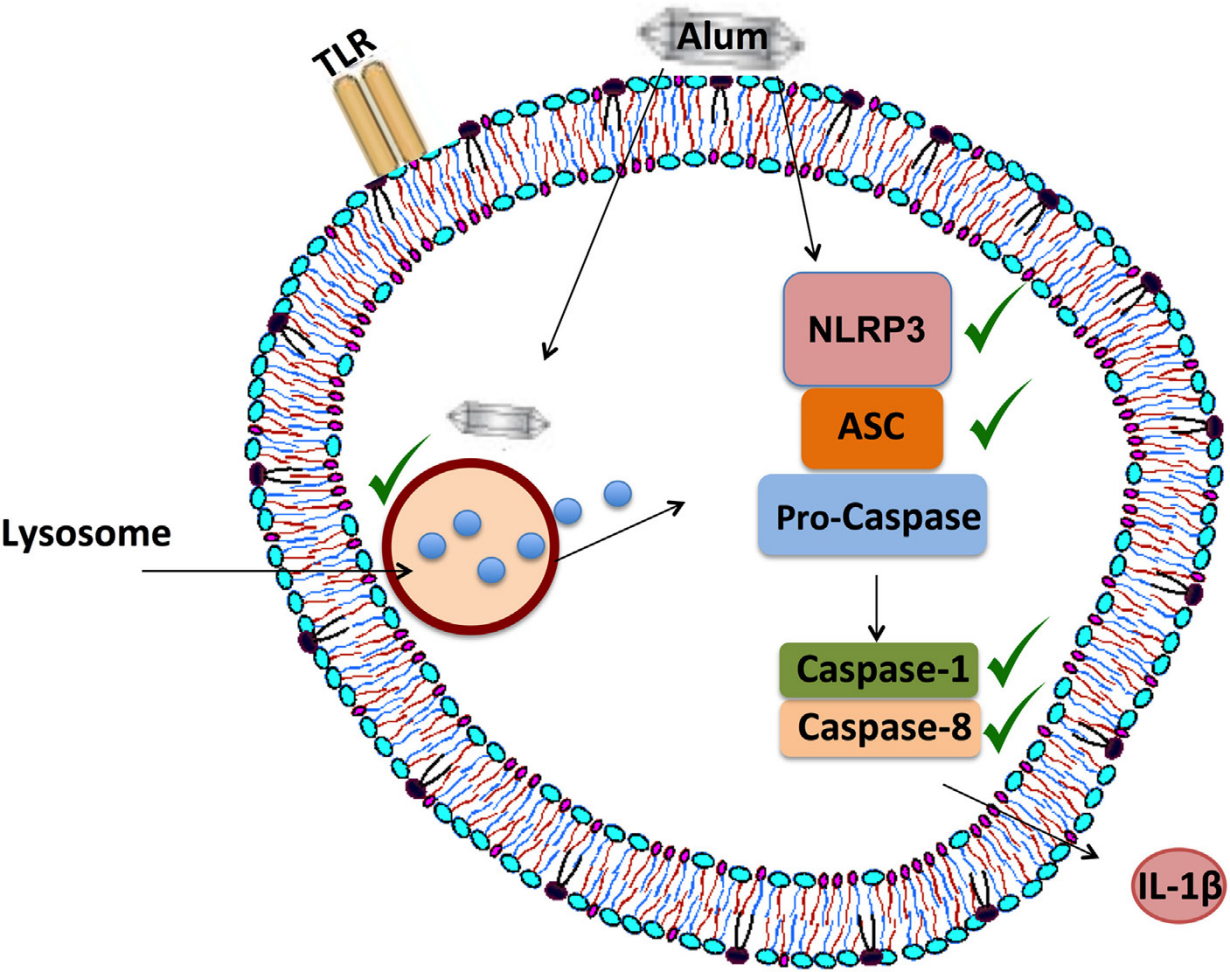
Proposed mechanism of alum-induced IL-1β secretion from bovine peripheral blood mononuclear cells (PBMCs). Alum enters the cell and induces lysosomal degradation to release cathepsins. From murine studies, it is proposed that cathepsin B can drive NLRP3 activation, which then mediates ASC oligomerization and caspase-1 and/or caspase-8 activation. These responses lead to the secretion of processed IL-1β from bovine PBMCs.

The intracellular processes underlying alum-induced IL-1β secretion were investigated, using inflammasome-specific inhibitors in cell culture. In mice, loss of NLRP3 and caspase-1 abrogates alum-induced IL-1β secretion *in vitro*, while a role for caspase-8 has also been proposed (8, 17). Consistent with what has been recorded in mice, alum-induced IL-1β secretion in bovine PBMCs is NLRP3 and caspase-1 dependent. Furthermore, results obtained using the FLICA assay confirm that alum can promote the activation of caspase-1 in bovine PBMCs. Unexpectedly, results from the FLICA assay showed that unstimulated PBMCs expressed active caspase-1. However, published data from Netea et al showed that caspase-1 was active in untreated human monocytes but not in macrophages (38). Alum-induced IL-1β secretion was reduced when caspase-8 was inhibited, thus suggesting that both caspase-1 and caspase-8 play roles in this process. From this study, it has been proposed that alum promotes IL-1β secretion by initiating the assembly of the NLRP3 inflammasome as outlined in Figure 8.

This research also established that alum promotes ASC oligomerization in bovine PBMCs. ASC mediates the interaction between NLRP3 and caspase-1, which ultimately leads to the formation of the inflammasome complex (39). Several studies have demonstrated that the oligomerization of ASC is key to NLRP3 inflammasome activation (22, 23), but this had not previously been addressed in bovine cells. Data from studies on human cells indicate that the ASC monomer is 22 kDa, while the oligomer is approximately 80–90 kDa (40, 41). In the current study, the size of the alum-induced ASC oligomers corresponds to what was recorded in humans. Similarly, in accordance with murine studies, these data also demonstrate that alum leads to lysosomal degradation and subsequent cathepsin release, which ultimately leads to the activation of the inflammasome in bovine PBMCs. It has been reported that cathepsin B mediates the activation of NLRP3 and caspase-1 leading to the secretion of processed IL-1β in mice (42–44).

To ensure the effectiveness of novel vaccine formulations, antigenicity has to be combined with adjuvanticity. Recent moves toward subunit vaccines yields higher specificity but with a requirement for effective adjuvants to activate and direct innate immunity and enhance immunogenicity (45). Although the role of alum in activating the inflammasome in mice has been well documented, its mechanism of action in cattle PBMCs was previously unclear. Given that alum has been incorporated into numerous bovine vaccine formulations, such as those against Leptospirosis, BVDV and Blue tongue virus, investigation of its mode of action in bovine PBMCs is important to facilitate more rational vaccine design. It will be important in future to address the role of the inflammasome in vaccine efficacy in a bovine context *in vivo* as there are conflicting reports from murine studies regarding the role of the NLRP3 inflammasome in driving adaptive immunity, particularly antibody responses (6, 46). The current data will inform future studies and aid the design of novel bovine vaccines aimed at targeting the inflammasome. As well as playing key roles in antigen presenting cells, inflammasome activation in epithelial cells could also be of significant importance in response to bovine mucosal vaccines.

## Ethics Statement

All animal procedures were carried out according to the provisions under the EU Protection of Animals Used for Scientific Purposes Regulations 2012 (SI No. 543 of 2012) as amended and Directive 2010/63/EU of the European Parliament issued from the Health Products Regulatory Authority Ireland—license number (AE 19132/P030). Human blood samples were collected from anonymous healthy blood donors from the Irish Blood Transfusion Service (IBTS) under license number (BI-AG-300919) issued from the School of Biochemistry and Immunology Research Ethics Committee, Trinity College Dublin in accordance with the Declaration of Helsinki.

## Author Contributions

CH conducted experiments, analyzed data and contributed to writing the article. AG and SMcC carried out experiments and participated in experimental design and data analysis. MC provided tools and participated in experimental design. AB provided advice with experimental design and data analysis. CS provided materials and participated in experimental design. KM codirected the study, provided samples, tools and participated in experimental design, data analysis, and writing the article. EL directed the study, participated in experimental design, data analysis, and writing the article.

## Conflict of Interest Statement

The authors declare that the research was conducted in the absence of any commercial or financial relationships that could be construed as a potential conflict of interest.
