# Supplementary material for: Alum Activates the Bovine NLRP3 Inflammasome
Source: Front Immunol. 2017 Nov 9;8:1494. doi: 10.3389/fimmu.2017.01494 (PMC5701618; doi:10.3389/fimmu.2017.01494)
Supplement: Supplementary file 1 [file image_1.pdf]

Supplementary Material

C. Harte[1, 2], A.L. Gorman[1], S. McCluskey[1], M. Carty[3], A.G. Bowie[3], C. Scott[4]., K.G. Meade[2] and E.C. Lavelle[1]

[1]Adjuvant Research Group, School of Biochemistry and Immunology, Trinity Biomedical Sciences Institute, Trinity College Dublin, Dublin, Ireland.
[2]Animal and Bioscience Research Department, Animal and Grassland Research and Innovation Centre, Teagasc, Grange, C15PW93, Co. Meath, Ireland.
[3]Viral Immune Evasion Group, School of Biochemistry and Immunology, Trinity Biomedical Sciences Institute, Trinity College Dublin, Dublin, Ireland
[4] Molecular Therapeutics, School of Pharmacy, Queen's University Belfast, 97 Lisburn Road, Belfast, BT9 7BL, UK.

***Correspondence:** lavellee@tcd.ie **or** kieran.meade@teagasc.ie

A

Unstimulated N=1-8

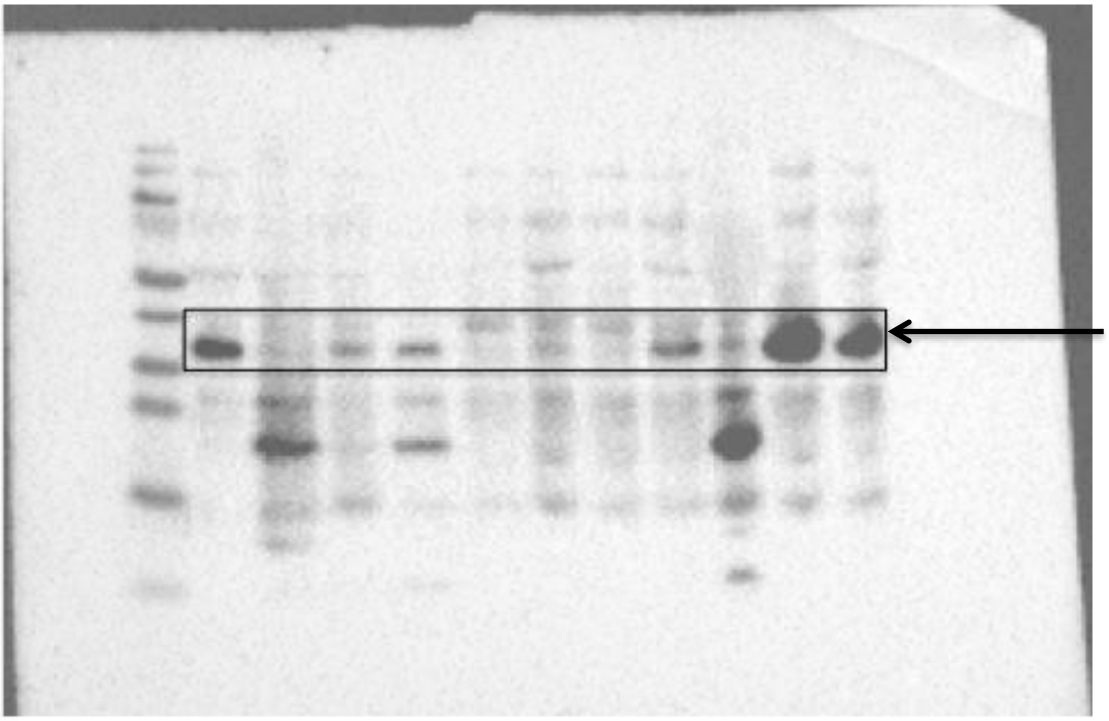

Pro-IL-1β 35-40 kDa

**LPS**	-	-	-	-	-	-	-	-	+	+
**Alum**	-	-	-	-	-	-	-	-	-	+

B

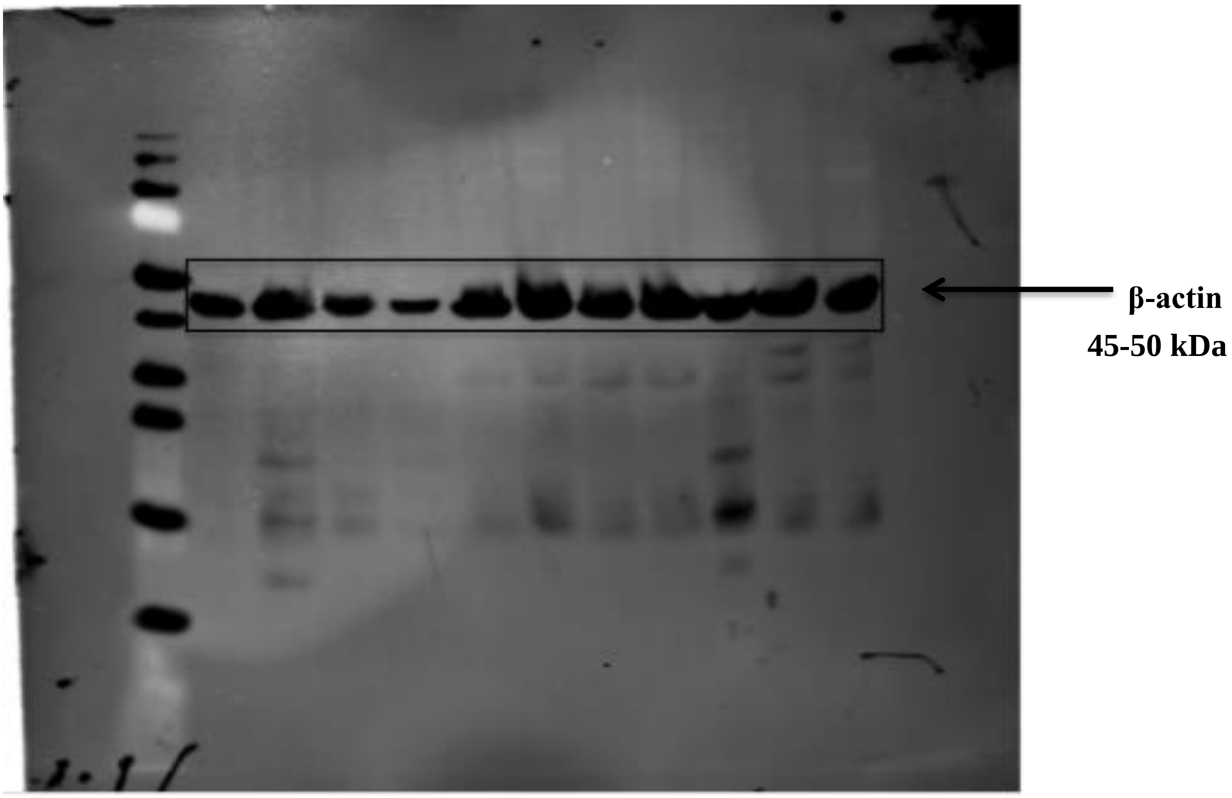

**LPS**	-	-	-	-	-	-	-	-	+	+
**Alum**	-	-	-	-	-	-	-	-	-	+

SUPPLEMENTARY FIGURE 1. Pre-formed IL-1β and β-actin present in bovine PBMCs. Bovine cells ($2X10^6$ cells/ml) from 8 animals were left unstimulated or treated with LPS and or/alum for 6 hours and analysed by western blot for pro-IL-1β (A). β-actin is evident in PBMCs of all 8 animals tested (B).

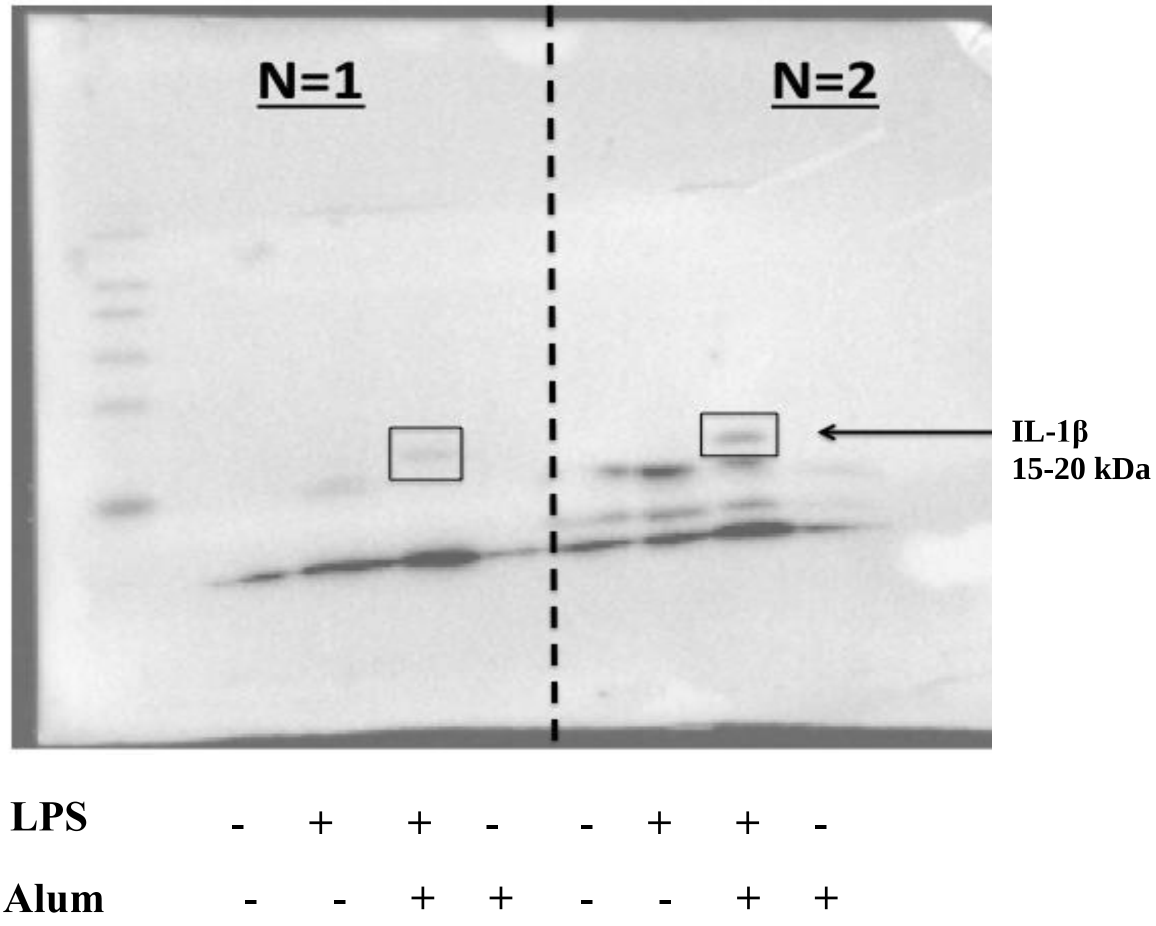

SUPPLEMENTARY FIGURE 2. Processed IL-1β is present in cell supernatants in response to LPS and alum. Secretory IL-1β is detected in the supernatants of bovine cells treated with LPS and alum.

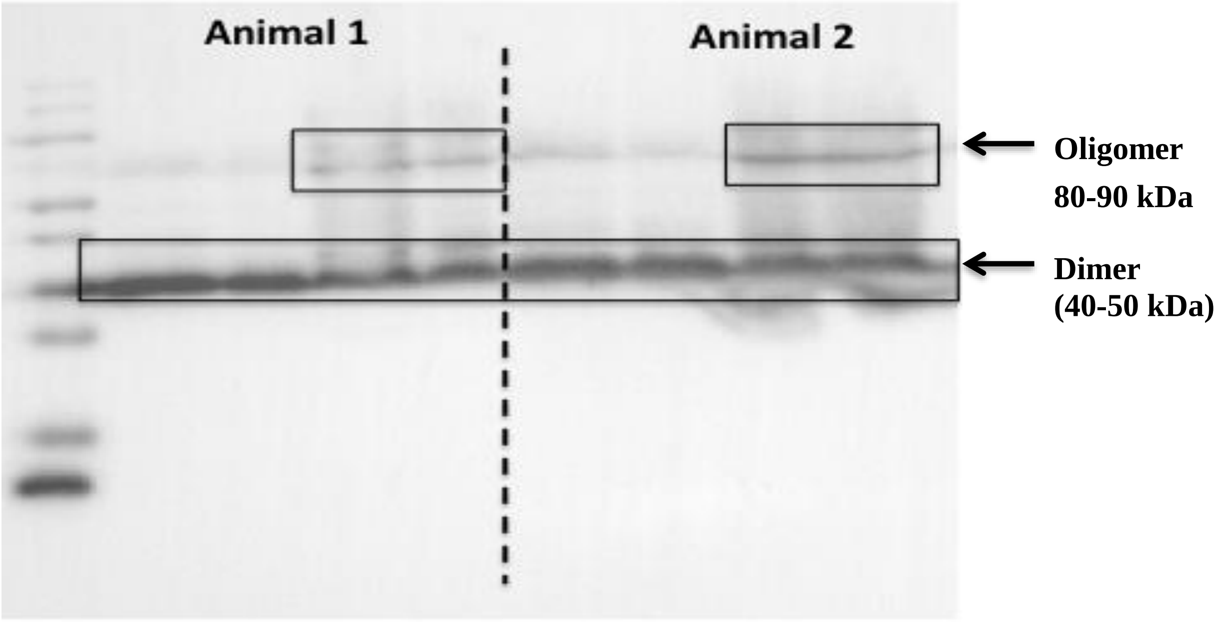

**LPS**	-	-	+	-	-	-	+	-
**Alum**	-	-	+	+	-	-	+	+

SUPPLEMENTARY FIGURE 3. Alum promotes the oligomerisation of ASC. Cells from 2 animals were stimulated with LPS 3 hours before alum was administered. Cell lysates were harvested 3 hours later and ASC oligomers (80-90 kDa) were detected through western blotting

**Molecular weights were determined using Thermo Scientific™ PageRuler™ Prestained 10-180kDa Protein Ladder*

Catalogue number: 11822124
